# 444. Effect of multidisciplinary management of CKD on proteinuria in people with HIV

**DOI:** 10.1093/ofid/ofac492.519

**Published:** 2022-12-15

**Authors:** Yussef Bennani, Lauren Richey, Hira Hasan, Annalisa Perez

**Affiliations:** LSU Health Sciences Center New Orleans, New Orleans, Louisiana; LSU Health Sciences Center New Orleans, New Orleans, Louisiana; LSU Health Sciences Center New Orleans, New Orleans, Louisiana; LSU Health Sciences Center New Orleans, New Orleans, Louisiana

## Abstract

**Background:**

Optimizing management of chronic kidney disease in people with HIV has become a more pressing priority both to minimize morbidity and preserve HIV antiretroviral options. Part of standard care is employing the beneficial effects of angiotensin converting enzyme (ACE) inhibitors and angiotensin II receptor blockers (ARB) in patients with clinically significant proteinuria. Our analysis looks at the proteinuria outcomes of PWH with CKD co-managed by a multidisciplinary team of infectious disease and nephrology physicians nested in a Ryan White-funded clinic in New Orleans.

**Methods:**

Patients with HIV and at least grade 3 CKD or with significant proteinuria were referred to the multidisciplinary clinic. Patients were evaluated by an HIV specialist and nephrologist at the initial visit for a jointly developed management plan. Renal function and proteinuria were measured at baseline and longitudinally per standard of care. Change in proteinuria and GFR over time were calculated. Patients were also assessed for whether they were prescribed ACE/ARB therapy, if appropriate. Patients included in this analysis were evaluated between January 1, 2021 and March 31, 2022.

**Results:**

During the analysis period, 46 patients with at least CKD 3 or proteinuria were evaluated. 35 patients were Black non-Hispanic, 9 were White non-Hispanic, and 2 were Hispanic. 39 were male, and 7 were female. 35 had a previous diagnosis of hypertension, and 10 had diabetes mellitus. Of the 42 with a recorded baseline urine protein measurement, 24 were found to have clinically significant proteinuria. Of those with proteinuria, 16 were on an ACE or ARB at their baseline evaluation. 20 were on ACE/ARB’s the end of the analysis period (p=0.058). Average baseline proteinuria was 1,158 mg/g, while average final was 938 mg/g, which was less but not significantly different (p=0.54). Mean baseline GFR was not significantly different from final GFR (50.8 mL/min vs 50.5 mL/min, p=0.67).

**Conclusion:**

A multidisciplinary model of managing CKD among people with HIV may improve adherence with management guidelines and limit progression of proteinuria and decrease in GFR. In general, the natural history of kidney disease with proteinuria is to worsen over time, so while not significant, proteinuria in this cohort did decrease over time

**Disclosures:**

**All Authors**: No reported disclosures.

